# The evaluation and management of corneal penetrating and perforating injuries during long-duration spaceflight

**DOI:** 10.1038/s41433-024-02996-4

**Published:** 2024-02-29

**Authors:** Alex Suh, Joshua Ong, Charles Robert Gibson, Thomas Mader, John Berdahl, Ethan Waisberg, Andrew G. Lee

**Affiliations:** 1 Tulane University School of Medicine, New Orleans, LA USA; 2 Department of Ophthalmology and Visual Sciences, University of Michigan Kellogg Eye Center, Ann Arbor, MI USA; 3 KBR, Houston, TX USA; 4 South Shore Eye Center, League City, TX USA; 5 NASA Ophthalmology Consultant, Moab, UT USA; 6 Vance Thompson Vision, Sioux Falls, SD USA; 7 Department of Ophthalmology, University of Cambridge, Cambridge, UK; 8 Center for Space Medicine, Baylor College of Medicine, Houston, TX USA; 9 Department of Ophthalmology, Blanton Eye Institute, Houston Methodist Hospital, Houston, TX USA; 10 The Houston Methodist Research Institute, Houston Methodist Hospital, Houston, TX USA; 11 Departments of Ophthalmology, Neurology, and Neurosurgery, Weill Cornell Medicine, New York, NY USA; 12 Department of Ophthalmology, University of Texas Medical Branch, Galveston, TX USA; 13 University of Texas MD Anderson Cancer Center, Houston, TX USA; 14 Texas A&M College of Medicine, Bryan, TX USA; 15 Department of Ophthalmology, The University of Iowa Hospitals and Clinics, Iowa City, IA USA

**Keywords:** Risk factors, Eye manifestations

The International Space Station (ISS) is one of the most remote and austere environments in which a human being can live and work. Responding to medical emergencies in such an environment can be extremely difficult. Although ISS crewmembers undergo extensive training for medical contingencies, the inherent complexities to evaluate and treat disease are daunting (e.g., limited diagnostic capabilities and medications, delayed access to medical professionals in needed specialties, time delay in communications). These challenges and barriers to access appropriate care can subject an ISS crewmember to risks of vision loss secondary to an ocular disorder or injury. The Lifetime Surveillance of Astronaut Health database has documented numerous ocular injuries throughout the history of the United States space program. This raises concerns about the potential for vision-threatening and mission-threatening ocular injuries on the ISS. Ocular, orbital, and cranial trauma (e.g., cabin contacts, crew member contact, elastic cords, secondary to chemical burns [1] or infection) have been reported on multiple ISS missions. Fortunately, to date none of the prior reported injuries have led to corneal perforation. However, these ocular scenarios emphasize the critical importance of addressing corneal injuries before escalation to perforations, especially with the commercialization of spaceflight [2, 3]. In this paper, we will discuss the use of onboard diagnostic tools and potential management modalities for the treatment of penetrating or perforating injuries to the cornea in spaceflight with current medications aboard the ISS.

## Risk factors

Corneal penetrating and perforating injuries can result from traumatic impact or conditions that cause corneal melting (e.g., microbial keratitis). Severe or poorly managed corneal abrasions, ulcers, and thermal burns are subject to infections that can progress to corneal perforations [4]. In the setting of microgravity, airborne particulates remain suspended (rather than falling to the floor on Earth) and can enter crewmembers’ eyes [3]. With NASA’s aim to return to the Moon and to travel to Mars, celestial debris will be an additional risk to ocular health. Lunar and Martian windstorms increase the likelihood of higher-velocity particles causing severe penetrating corneal injuries [5]. Recent literature has described 70 corneal abrasions, 6 chemical burns and 5 ocular infections in a review of NASA Space Shuttle and ISS missions [1]. These were superficial corneal injuries that were successfully treated with topical antibiotics. In contrast, future longer-duration crewed missions to Mars and beyond might require more innovative and novel technology-driven platforms to address the risks for corneal perforation. We review the literature and propose potential countermeasures to prepare for such contingencies for future long term space exploration.

## Diagnostic techniques

### Medical management

A penetrating corneal injury enters the cornea, but a perforating corneal injury is a through and through injury with an entrance and exit wound. Crews without a medical doctor will assign a designated medical officer, who undergoes 40 h of paramedic-level training (e.g., administering injections, suturing wounds, eye-washing protocols) [6]. In the event of a severe penetrating corneal injury, crewmembers are advised not to disturb the offending object or put any pressure on the globe from rubbing or patching. Rather, they should gently apply a rigid eye shield over the injured eye to avoid accidental contact [7]. It is crucial to obtain a detailed history of precisely how the injury occurred as this may help to determine the degree of injury severity (e.g., Was the injury caused by a high velocity particle originating from a burst mechanism or from floating debris in the cabin?). The trained crew on the ISS can utilize magnified external ocular photography, the onboard portable ocular ultrasound, and Heidelberg Spectralis OCT2 imaging of the anterior and posterior segment to accurately detect the presence, location, and composition of intraocular foreign bodies with high sensitivity and specificity [8]. Remote guidance is available by the crew flight surgeon and subject matter experts at NASA’s Johnson Space Center to develop a diagnosis and implement vision-saving interventions.

Depending on severity of penetration, microsurgical repair and systemic antibiotics may be indicated. The ISS is equipped with a comprehensive array of medications, comparable to those used in terrestrial management of corneal perforations, capable of minimizing the risk of infection and offering therapeutic relief [9, 10]. Topical anaesthetics (e.g., tetracaine (0.5%, 15 mL)), cycloplegic drops (e.g., cyclopentolate (Cyclogyl) 2%, 15 mL; tropicamide (Mydriacyl) 1%, 15 mL), and anti-inflammatory and analgesic agents (e.g., ketorolac (Toradol) 30 mg/mL, 2 mL; acetaminophen (Tylenol) 325 mg; Aspirin 325 mg; Ibuprofen (Motrin) 400 mg) may provide relief during the painful ocular injury.

Prophylactic broad-spectrum antibiotic ointment is available to reduce the potential of secondary infection. Topical medications **(**Erythromycin ointment (0.5%); Moxifloxacin (Vigamox, 0.5%); Tobramycin and Dexamethasone (Tobradex), 0.3%; 0.1%) are currently available on the ISS [4, 11]. Among the other antibiotics aboard, doxycycline (Vibramycin, 100 mg) has a high lipid solubility, providing stronger penetration into the aqueous humour of the cornea [12]. Vitamin C supplementations have also demonstrated accelerated recovery and healing properties for corneal injuries [13]. For more severe cases, temporizing cyanoacrylate glue is present on the ISS. Although the use of glue is not recommended for large or more severe (open globe) ocular injuries, the glue can serve as a viable temporizing option for immediate management of small, but severe corneal perforations (<3 mm) to seal the wound [14]. Although most ISS injuries have resulted in small superficial corneal wounds, embedded corneal foreign bodies have the potential to cause localized corneal necrosis and dislodgement from the cornea with subsequent aqueous leakage and loss of anterior chamber volume [15]. Thus, all corneal injuries require very precise documentation and follow up. It is anticipated that astronauts with severe perforating corneal injuries will likely require transport to Earth for appropriate long-term care. However, the routes of management described in this correspondence are appropriate for the initial management of even severe injuries on the ISS. Artificial tears (e.g., Carboxymethylcellulose (Refresh Plus, 0.5%); Hypromellose (Nature’s Tears, 0.4%)); Mineral Oil and White Petrolatum (Refresh PM, 42.5%; 57.3%, 3.5 gm) may assist with symptomatic management [16].

Future missions should consider incorporating contact bandage lenses or collagen corneal shields to provide a more conducive environment for epithelial and stromal healing and effective topical drug delivery [17]. They can also be applied following glue application to provide an additional layer of comfort and support. These treatment methods are effective but require detailed training prior to application. Depending on mission timelines and injury severity, additional treatment options may be considered upon abort to Earth, including amniotic membrane or corneal transplantation [18].

### Celestial missions and evacuation

The Crew Return Vehicle (CRV) is a dedicated lifeboat or escape module for the ISS, currently fulfilled by the Soyuz spacecraft or SpaceX’s Crew Dragon. In the event of an emergency medical return, the CRV may be separated from the ISS in under three minutes and autonomously return to Earth’s surface in approximately three hours [19]. Usually two CRVs, which can hold approximately three crewmembers each, are simultaneously docked to the ISS. If a penetrating eye injury were to warrant prompt evacuation, half of the crewmembers would accompany the injured member back to Earth for more comprehensive medical treatment [20].

Unlike ISS missions, lunar sortie (human spaceflight to the Moon), lunar outpost (moonbase establishment), and Near-Earth (NEA) missions will not have possible evacuation back to Earth [7]. Moreover, communication latencies (~20 min to and from Mars) between flight surgeons on Earth will further complicate medical decision-making processes [21]. In the advent of the rapid development of machine learning, telemedicine may be supplemented with artificial intelligence when determining the next best step in management for penetrating corneal injuries, especially when communication latencies delay immediate intervention in the setting of medical emergencies [22].
